# Magnitude-constrained optimal chaotic desynchronization of neural populations

**DOI:** 10.3389/fnetp.2025.1646391

**Published:** 2025-10-21

**Authors:** Michael Zimet, Faranak Rajabi, Jeff Moehlis

**Affiliations:** ^1^ Interdepartmental Graduate Program in Dynamical Neuroscience, University of California, Santa Barbara, Santa Barbara, CA, United States; ^2^ Department of Mechanical Engineering, University of California, Santa Barbara, Santa Barbara, CA, United States

**Keywords:** optimal control, desynchronization, deep brain stimulation, Lyapunov exponent, network physiology

## Abstract

In this paper, we calculate magnitude-constrained optimal stimuli for desynchronizing a population of neurons by maximizing the Lyapunov exponent for the phase difference between pairs of neurons while simultaneously minimizing the energy which is used. This theoretical result informs the way optimal inputs can be designed for deep brain stimulation in cases where there is a biological or electronic constraint on the amount of current that can be applied. By exploring a range of parameter values, we characterize how the constraint magnitude affects the Lyapunov exponent and energy usage. Finally, we demonstrate the efficacy of this approach by considering a computational model for a population of neurons with repeated event-triggered optimal inputs.

## 1 Introduction

As deep brain stimulation (DBS) emerges as an effective therapy for a wide range of neurological disorders, theoretical perspectives are growing to help inform effective stimulation protocols. DBS technology involves surgically implanting electrodes to deliver electrical stimuli over time to specific brain regions with wires connecting the implanted electrodes to an implantable pulse generator, which can be programmed to set DBS parameters (Lozano and Lipsman, 2013; Sandoval-Pistorius et al., 2023; Frey et al., 2022). Novel technologies enable the optimization of DBS stimulation parameters including the input pulse’s shape, amplitude, frequency, and interstimulus interval (Najera et al., 2023; Krauss et al., 2021). Computational analysis of the interaction between an applied electrical stimulus and the spiking dynamics of neural populations can inform the effective design of DBS parameters to enhance clinical outcomes while respecting device engineering constraints.

Among other conditions, deep brain stimulation has become an effective treatment for Parkinson’s disease, where pathological synchronization of the basal ganglia-cortical loop is associated with dopaminergic denervation of the striatum, which over time leads to motor impairment including tremors, bradykinesia, and akinesia (Hammond et al., 2007; Neumann et al., 2007). In particular, the parkinsonian low-dopamine state is observed to be related to excessive synchronization in the beta frequency band (15–30 Hz) in the subthalamic nucleus (STN) of the basal ganglia (Rubchinsky et al., 2012; Asl et al., 2022). It has been proposed that the symptoms of parkinsonian resting tremors are caused by excessive synchronization in populations of neurons firing at a similar frequency to that of the tremor (Tass, 2006). Deep brain stimulation works as a therapeutic intervention by modulating such synchronization patterns, often ameliorating motor impairment for patients living with Parkinson’s disease. DBS is also used in the treatment of essential tremor (ET), epilepsy, Tourette’s syndrome, obsessive compulsive disorder, and treatment-resistant depression.

The most commonly offered protocol for DBS therapy is continuous high-frequency stimulation (Lozano and Lipsman, 2013; Sandoval-Pistorius et al., 2023). Despite its clinical success, conventional high-frequency DBS faces several limitations including diminishing efficacy over time, stimulation-induced side effects, and high energy consumption necessitating battery replacements. Furthermore, as open-loop stimulation, where the device continuously applies electrical inputs as long as it is on, its static parameters do not adapt to the dynamic nature of disease symptoms, limiting its long-term effectiveness. In recent years, there has been growing interest in developing alternative stimulation paradigms that can effectively disrupt pathological synchrony while minimizing energy consumption and side effects. Several desynchronization methods have been proposed and tested on patients, including coordinated reset stimulation (Tass, 2003; Manos et al., 2021), adaptive deep brain stimulation (aDBS) (Sandoval-Pistorius et al., 2023), and phase-specific stimulation approaches (Cagnan et al., 2017). Such techniques often leverage theoretical frameworks from nonlinear dynamics and control theory to design stimuli that can efficiently desynchronize neural populations.

In particular, coordinated reset uses multiple electrode implants which deliver identical impulses separated by a time delay between implants (Tass, 2003; Lysyansky et al., 2011; Lücken et al., 2013; Popovych and Tass, 2014; Kubota and Rubin, 2018; Khaledi-Nasab et al., 2022). This leads to clustering behavior for the neural populations, in which each cluster fires at different times, giving (partial) desynchronization of the dynamics. This approach has achieved preliminary clinical success (Adamchic et al., 2014; Manos et al., 2021). In adaptive deep brain stimulation (aDBS), closed-loop systems monitor biomarkers in real time and can initiate changes in stimulation parameters (Sandoval-Pistorius et al., 2023; Oehrn et al., 2024). The goal of aDBS is to improve clinical outcomes by designing control signals based on neural data to deliver stimulation only when needed. For instance, in Parkinson’s disease, a potential biomarker is the amplitude of the pathological beta rhythms, with stimulation becoming active if this exceeds some prescribed threshold. On demand stimulation through aDBS can prolong device battery life, thereby extending device lifetimes and prolonging the interval between surgeries in clinical treatment protocols. Finally, for phase-specific stimulation the inputs occur at a particular dynamical phase in order to disrupt synchrony (Cagnan et al., 2017; Cagnan et al., 2019; cf. Holt et al., 2016).

In parallel, there have been a number of computational studies exploring the mechanisms by which DBS might be working (e.g., Santaniello et al., 2015; Spiliotis et al., 2022). There have also been theoretical and computational studies exploring different strategies for desynchronizing neural populations (Wilson and Moehlis, 2022), with approaches including delayed feedback control (Rosenblum and Pikovsky, 2004a; Rosenblum and Pikovsky, 2004b; Popovych et al., 2006; Popovych et al., 2017), phase randomization through optimal phase resetting (Danzl et al., 2009; Nabi et al., 2013; Rajabi et al., 2025), phase distribution control (Monga et al., 2018; Monga and Moehlis, 2019), cluster control (Wilson and Moehlis, 2015a; Matchen and Moehlis, 2018; Wilson, 2020; Qin et al., 2023), machine learning and data-driven approaches (Matchen and Moehlis, 2021; Vu et al., 2024), and chaotic desynchronization (Wilson et al., 2011; Wilson and Moehlis, 2014b; Wilson and Moehlis, 2014a). Each of these approaches has advantages and disadvantages based on the control objective and what is known about and what can be measured for the neural dynamics (Wilson and Moehlis, 2022).

In this paper, we focus on chaotic desynchronization, for which an energy-optimal stimulus exponentially desynchronizes a population of neurons. This approach relies on phase reduction methods, which have proven particularly valuable in analyzing and controlling neural oscillators (Monga et al., 2019; Wilson and Moehlis, 2022). These methods allow for the simplification of complex neuronal dynamics into phase models, where the behavior of an oscillating neuron can be characterized by its phase and response to perturbations, captured by the phase response curve (PRC). Unlike previous studies of chaotic desynchronization, here we include a constraint on stimulus magnitude. Such constraints are important engineering considerations for the practical applications of DBS, as there can exist both biological limitations on the maximum electrical stimulation that can be safely applied to brain tissue as well as electronic limitations on the current that stimulation devices can reliably store and deliver over time.

Specifically, in this paper we calculate magnitude-constrained optimal stimuli that maximize the Lyapunov exponent for the phase difference between pairs of neurons while simultaneously minimizing energy consumption. The Lyapunov exponent quantifies the exponential divergence rate of initially close trajectories, making it an appropriate measure of desynchronization efficiency. By systematically exploring different constraint magnitudes, we characterize the tradeoff between maximum allowable stimulus amplitude, desynchronization efficacy, and energy usage. We set up the optimal control problem in Section 2.1. In Section 2.2 we describe several canonical phase response curves representing different types of neuronal dynamics: Sinusoidal, SNIPER, Hodgkin-Huxley, and Reduced Hodgkin-Huxley models. In Section 3.1, we investigate our approach for each PRC, computing the optimal stimulus under various magnitude constraints and evaluating its performance in desynchronizing initially synchronized neurons. In Section 3.2, we validate our approach using computational simulations of neural populations with coupling and noise, demonstrating that our magnitude-constrained optimal stimuli can effectively desynchronize neural populations. Finally, a discussion of our results is given in Section 4. Overall, this work provides a theoretical foundation for designing energy-efficient DBS protocols that respect hardware and biological constraints while effectively disrupting pathological neural synchronization. We respectfully present this study as a tribute to the pioneering work of Hermann Haken on the control of complex systems.

## 2 Methods

### 2.1 Optimal control problem

We present a procedure for finding an energy-optimal stimulus which maximizes the Lyapunov exponent associated with the phase difference between a pair of neurons, while accounting for a constraint on the stimulus magnitude. This approach is based on the phase reduction of neural oscillators in the presence of an input (see, for example, Monga et al., 2019), and only requires knowledge of a neuron’s phase response curve (PRC). We note that the PRC can in principle be measured experimentally (Netoff et al., 2012), or can be calculated numerically if the model is known (Ermentrout, 2002; Monga et al., 2019). In particular, we consider the following set of equations:

$$\frac{d\theta_{i}}{dt}=\omega +Z\left(\theta_{i}\right)u\left(t\right),\;i=1,2,$$ (1)

where $\theta_{i}\in \left.\left[0,2\pi \right.\right)$ is the phase of the $i^{th}$ neuron; ω=2π/T , where T is the period of the neuron in the absence of stimulus; and u(t) is the control stimulus. Note that here we are assuming that the neurons are identical (having the same ω and Z(⋅) ), and for simplicity we assume that the neurons are the same distance from the electrode so they receive the same stimulus u(t) . Neuron i fires an action potential when $\theta_{i}$ crosses through 0.

Following Wilson and Moehlis (2014b), we suppose that the neurons are nearly synchronized $\left(\theta_{1}\approx \theta_{2}\right)$ . Defining $\phi =\theta_{2}-\theta_{1}$ , we obtain

$$\frac{d\phi }{dt}=Z^{\prime }\left(\theta \right)u\left(t\right)\phi +O\left(\phi^{2}\right).$$ (2)

Linearizing about ϕ=0 , the solution to Equation 2 is $\phi ∼e^{\Lambda t}$ , where

$$\Lambda \left(\tau \right)=\frac{\log\left(\phi \left(\tau \right)\right)}{\tau }=\frac{\int_{a}^{a+\tau }Z^{\prime }\left(\theta \left(s\right)\right)u\left(s\right)ds}{\tau }.$$ (3)

Here Λ can be viewed as the finite time Lyapunov exponent (Abouzeid and Ermentrout, 2009), which characterizes the exponential growth or decay of the phase difference ϕ . A positive Lyapunov exponent will correspond to the divergence of nearby trajectories, and hence desynchronization. We note that this Lyapunov exponent corresponds to phase difference direction, so it is not directly related to the non-trivial Floquet multipliers which describe transverse stability of the periodic orbits for the neurons (Guckenheimer and Holmes, 1983). We formulate the control problem in terms of the cost function

$$G\left[u\left(t\right)\right]=\int_{0}^{t_{1}}\left\{u{\left(t\right)}^{2}-\beta Z^{\prime }\left(\theta \right)u\left(t\right)\right\}dt,$$ (4)

where the goal is to maximize the Lyapunov exponent while minimizing the energy used, where the energy is the integral of the square of the control stimulus u . Here $t_{1}$ is the time that we choose for the duration of the stimulus, β>0 is a parameter that scales the importance of the Lyapunov exponent term relative to the energy term. Generalizing the formulation in Wilson and Moehlis (2014b), here we consider a magnitude constraint on the control stimulus given by Equation 5:

$$|u\left(t\right)|\leq u_{\max}.$$ (5)

In order to account for this constraint, we use a Hamiltonian formulation for the optimal control problem (Kirk, 1998), with the Hamiltonian given in Equation 6:

$$H\left(\theta ,\lambda ,u\right)=u^{2}-\beta Z^{\prime }\left(\theta \right)u\left(t\right)+\lambda \left(\omega +Z\left(\theta \right)u\left(t\right)\right),$$ (6)

where λ is the Lagrange multiplier or co-state for the system. From Hamilton’s equations,

$$\dot{\theta }=\frac{\partial H}{\partial \lambda }=\omega +Z\left(\theta \right)u\left(t\right),$$ (7)

$$\dot{\lambda }=-\frac{\partial H}{\partial \theta }=\left[\beta Z^{\prime \prime }\left(\theta \right)-\lambda Z^{\prime }\left(\theta \right)\right]u\left(t\right).$$ (8)

This defines a two-point boundary value problem which must be solved subject to the boundary conditions θ(0)=0 and $\theta \left(t_{1}\right)=\omega t_{1}$ . The latter boundary condition ensures that the phase at time $t_{1}$ is the same as what it would’ve been in the absence of stimulus. The function u(t) in these equations will be found using Pontryagin’s minimum principle (Kirk, 1998), which states that u should be chosen as the extremum of the Hamiltonian, subject to the constraints. If there is no constraint on the magnitude of u(t) , the optimal control stimulus is the solution to ∂H/∂u=0 , giving Equation 9:

$$2u-\beta Z^{\prime }\left(\theta \right)+\lambda Z\left(\theta \right)=0$$ (9)

$$\Rightarrow u\left(t\right)=\frac{\beta Z^{\prime }\left(\theta \right)-\lambda Z\left(\theta \right)}{2}\equiv \tilde{u}\left(t\right),$$ (10)

where $\tilde{u}\left(t\right)$ is the optimal unconstrained input. With constraints, Pontryagin’s minimum principle gives the following expression for the optimal magnitude-constrained input $u^{*}\left(t\right)$ :

$$u^{*}\left(t\right)=\left\{\begin{array}{cc} u_{max}\; & if\;\tilde{u}\left(t\right)\geq u_{max},\\ \tilde{u}\left(t\right)\; & if\;-u_{max}<\tilde{u}\left(t\right)<u_{max},\\ -u_{max}\; & if\;\tilde{u}\left(t\right)\leq -u_{max}. \end{array}\right.$$ (11)

In particular, the optimal $u^{*}\left(t\right)$ might or might not be the solution to ∂H/∂u=0 , because the extremum may be reached at a constraint boundary. To summarize, we solve the two-point boundary value problem Equations 7, 8 using Equation 11. This is done numerically using a shooting method, and the optimal stimulus is given by Equation 11.

### 2.2 Example phase response curves

**FIGURE 1:**
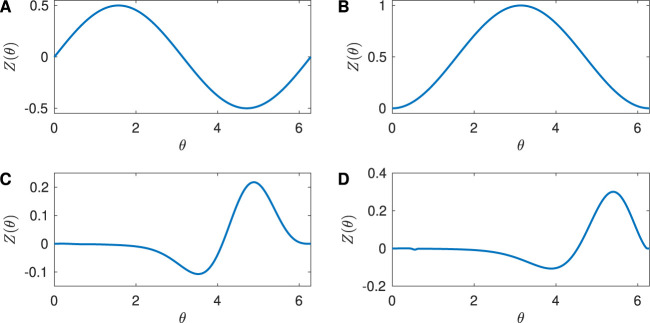
Phase Response Curves for the neuron models considered in the paper. **(A)** Sinusoidal with $Z_{d}=0.5$ , **(B)** SNIPER with $Z_{d}=0.5$ , **(C)** Hodgkin-Huxley, and **(D)** Reduced Hodgkin-Huxley.

The PRC quantifies the effect of an external stimulus on the phase of a periodic orbit. In this paper, we consider four example PRCs: Sinusoidal, SNIPER, Hodgkin-Huxley, and Reduced Hodgkin-Huxley, as shown in Figure 1.

The Sinusoidal PRC

$$Z\left(\theta \right)=Z_{d}\sin\theta ,$$ (12)

shown in Figure 1A with $Z_{d}=0.5$ , is a special case of the PRC which is found for periodic orbits close to a supercritical Hopf bifurcation. (Recall that when a parameter is on one side of a supercritical Hopf bifurcation there is a stable fixed point and no periodic orbit, and when the parameter is on the other side of the supercritical Hopf bifurcation there is an unstable fixed point and a stable periodic orbit). More generally, the PRC for a periodic orbit close to a supercritical Hopf bifurcation is a phase-shifted form of the PRC (Equation 12); see Brown et al. (2004). This is an example of a Type II PRC (Hansel et al., 1995), in which the PRC takes both positive and negative values.

Periodic orbits can also arise from a SNIPER bifurcation, which stands for Saddle-Node Infinite Period bifurcation; this is also often called a SNIC bifurcation, which stands for Saddle-Node Invariant Circle bifurcation. Here, for a parameter on one side of the bifurcation there is a stable fixed point and a saddle fixed point that lie on an invariant circle. As the parameter is varied, these fixed points annihilate in a saddle-node bifurcation, and when the parameter is on the other side of the bifurcation there is a stable periodic orbit whose period approaches infinity as the bifurcation is approached. For a periodic orbit near a SNIPER bifurcation, the PRC is approximately given by Equation 13 (Ermentrout, 1996; Brown et al., 2004):

$$Z\left(\theta \right)=Z_{d}\left(1-\cos\theta \right),$$ (13)

shown in Figure 1B with $Z_{d}=0.5$ . This is an example of a Type I PRC (Hansel et al., 1995; Ermentrout, 1996), in which the PRC takes only non-negative values.

The Hodgkin-Huxley equations are a well-studied conductance-based model for neural activity, and were developed to describe the dynamics for a squid giant axon (Hodgkin and Huxley, 1952). Mathematically, they are a four-dimensional set of coupled ordinary differential equations for the voltage across the neural membrane and three gating variables associated with the flow of ions across the membrane. The full equations are given in the Supplementary Appendix. We chose a baseline current value $I_{b}=10$ so that the Hodgkin-Huxley equations have a stable periodic orbit, and then found the PRC for this periodic orbit using XPP (Ermentrout, 2002). For computational convenience, we approximate this as a Fourier series with the first ten sin(⋅) and cos(⋅) terms to give the PRC shown in Figure 1C.

Finally, the Reduced Hodgkin-Huxley equations are an approximation to the full Hodgkin-Huxley equations (Keener and Sneyd, 1998; Moehlis 2006). Mathematically, they are two-dimensional set of coupled ordinary differential equations for the voltage across the neural membrane and one gating variable. The equations are given in the Supplementary Appendix. We chose a baseline current value $I_{b}=10$ so that the Reduced Hodgkin-Huxley equations have a stable periodic orbit, and then found the PRC for this periodic orbit using XPP (Ermentrout, 2002). For computational convenience, we approximate this as a Fourier series with the first two hundred sin(⋅) and cos(⋅) terms to give the PRC shown in Figure 1D. The PRCs for the Hodgkin-Huxley and Reduced Hodgkin-Huxley models are examples of Type II PRCs.

## 3 Results

### 3.1 Results for pairs of neurons

In this section, we consider the dynamics of a pair of neurons satisfying Equation 1, where $u^{*}\left(t\right)$ is chosen to be the optimal control stimulus for different values of the constraint $u_{max}$ . For simplicity, we will take $t_{1}=T$ , so that the duration of the control stimulus is equal to the period of the neuron in the absence of stimulus. By design, we expect that application of one cycle of the optimal stimulus will cause the phase difference ϕ to increase, at least when the initial value for ϕ is small. We will consider an event-based approach for which multiple cycles of the optimal control stimulus are applied, where a new cycle of the control stimulus is triggered when $\theta_{1}=0$ , that is, when Neuron 1 fires an action potential. We will see that this leads to growing phase difference ϕ .

**FIGURE 2:**
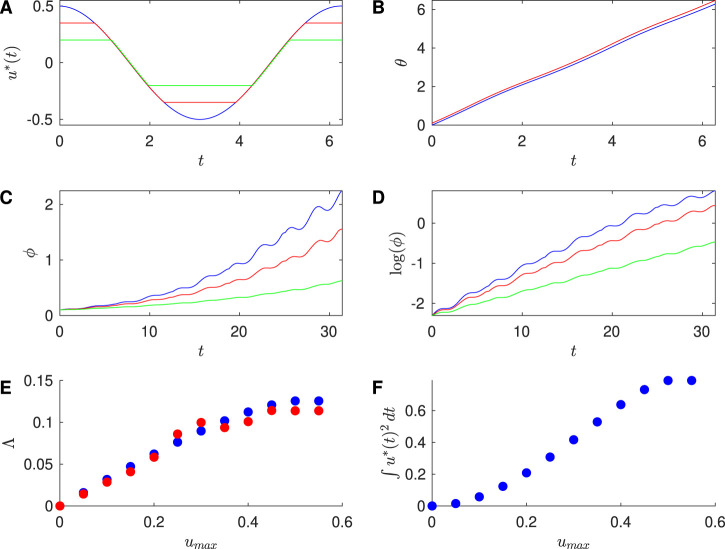
Results for Sinusoidal PRC, with $Z_{d}=0.5$ , ω=1 , and β=2 . **(A)** Optimal stimulus calculated for the unconstrained case (blue), $u_{max}=0.35$ (red), and $u_{max}=0.2$ (green). **(B)** Time series traces of the phases $\theta_{1}$ (blue) and $\theta_{2}$ (red) of two neurons, where both have the optimal stimulus applied with $u_{max}=0.35$ . **(C)** Phase difference ϕ between two neurons over multiple stimuli. Each stimulus $u^{*}$ is triggered when $\theta_{1}=0$ . **(D)** Log of the difference ϕ over multiple stimuli. **(E)** Approximation to the Lyapunov exponent $\Lambda_{fit}$ found from the slopes of the fits to log(ϕ) versus t (red) and the Lyapunov exponent $\Lambda_{calc}$ (blue), for different values of $u_{max}$ . **(F)** Effect of the constraint on the energy used for the optimal stimulus, here computed for one cycle of the stimulus. For panels **(B–F)**, the initial conditions are $\theta_{1}=0$ and $\theta_{2}=0.1$ .

Figure 2 shows results for the Sinusoidal PRC with $Z_{d}=0.5$ , β=2 and ω=1 , corresponding to T=2π . In particular, Figure 2A shows the calculated optimal control stimuli for the unconstrained case, $u_{max}=0.35$ , and $u_{max}=0.2$ . In this case, adding a magnitude constraint gives an optimal control stimulus which appears to be very similar to the unconstrained stimulus “chopped off” at the constraint; we will discuss this further below. We observe that the unconstrained input has the highest efficacy of desynchronization as measured by the Lyapunov exponent, and also the highest energy utilization. The input with a constraint $u_{max}=0.35$ gives desynchronization results that are similar while achieving a significant reduction in energy usage.

To investigate the efficacy of desynchronization between Neurons 1 and 2, we ran simulations of the phases of Neurons 1 and 2 over a full cycle of the control stimulus, with initial conditions $\theta_{1}=0$ and $\theta_{2}=0.1$ . Figure 2B shows the time series traces of these two neurons for the Sinusoidal PRC. We see growing desynchronization over one cycle of the control stimulus, as measured by the phase difference between the two traces.

Next, we apply successive control stimuli to pairs of neurons with the event-based approach described above. We compute the phase difference ϕ between the pair of neurons, which is observed to grow exponentially over multiple cycles of the control stimulus; see Figure 2C. This is also evident in Figure 2D, where we observe that log(ϕ) approximately grows linearly with t . To quantify this, we estimate the Lyapunov exponent based on the line of best fit to log(ϕ) versus t over multiple event-triggered control stimuli. We used the first half of the time interval for these fits, since there is saturation in these traces in the later part of time interval as the small ϕ approximation used to obtain Equation 3 no longer holds. These estimated Lyapunov exponents $\Lambda_{fit}$ are plotted at varying values of the constraint in Figure 2E, along with estimates $\Lambda_{\mathrm{calc}}$ obtained by plugging the computed stimulus into Equation 3 and numerically evaluating the integral over one cycle of the stimulus according to Equation 14:

$$\Lambda_{calc}\equiv \frac{1}{T}\int_{0}^{T}Z^{\prime }\left(\theta \left(s\right)\right)u^{*}\left(s\right)ds.$$ (14)

Table 1 compares $\Lambda_{fit}$ and $\Lambda_{\mathrm{calc}}$ for several values of $u_{max}$ ; good agreement is found between these approaches. Finally, Figure 2F shows the energy

$$\int_{0}^{T}{\left[u^{*}\left(t\right)\right]}^{2}dt$$

used over one cycle of the control stimulus for a range of values of $u_{\max}$ .

**TABLE 1:** For the Sinusoidal PRC, comparison of the Lyapunov exponent $\Lambda_{fit}$ estimated from slope of the line of best fit for log(ϕ) versus t with the Lyapunov exponent $\Lambda_{calc}$ calculated from the integral formulation.

$u_{max}$	$\Lambda_{fit}$	$\Lambda_{\mathrm{calc}}$
0.5	0.114	0.126
0.35	0.0937	0.102
0.2	0.0583	0.0621

**FIGURE 3:**
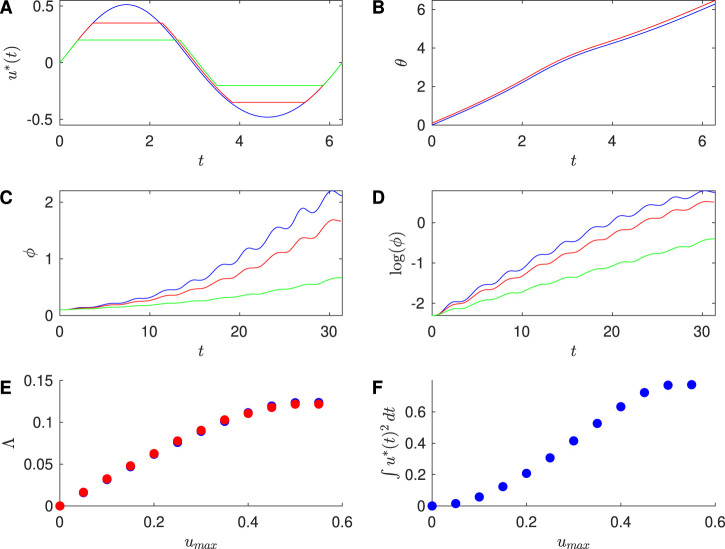
Results for SNIPER PRC with $Z_{d}=0.5,\omega =1$ , and β=2 . **(A)** Optimal stimulus calculated for the unconstrained case (blue), $u_{max}=0.35$ (red), and $u_{max}=0.2$ (green). **(B)** Time series traces of the phases $\theta_{1}$ (blue) and $\theta_{2}$ (red) of two neurons, where both have the optimal stimulus applied with $u_{max}=0.35$ . **(C)** Phase difference ϕ between two neurons over multiple stimuli. Each stimulus $u^{*}$ is triggered when $\theta_{1}=0$ . **(D)** Log of the difference ϕ over multiple inputs. **(E)** Approximation to the Lyapunov exponent $\Lambda_{fit}$ found from the slopes of the fits to log(ϕ) versus t (red) and the Lyapunov exponent $\Lambda_{\mathrm{calc}}$ (blue), for different values of $u_{max}$ . **(F)** Effect of the constraint on the energy used for the optimal stimulus, here computed for one cycle of the stimulus. For panels **(B–F)**, the initial conditions are $\theta_{1}=0$ and $\theta_{2}=0.1$ .

**FIGURE 4:**
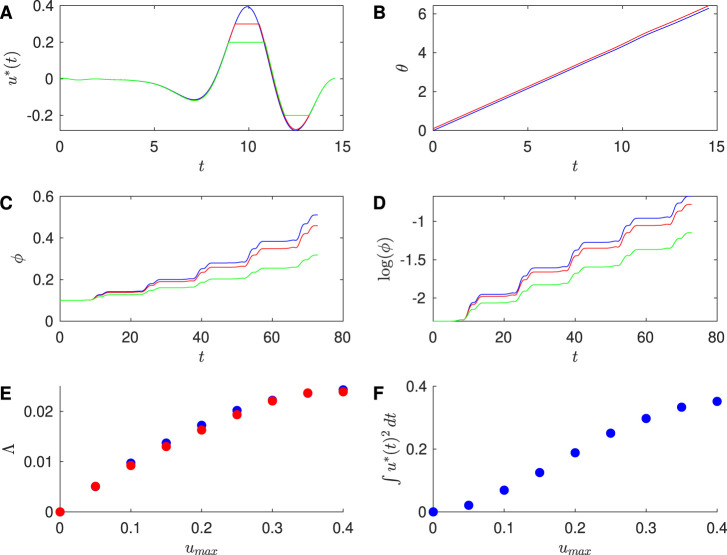
Results for Hodgkin-Huxley PRC with β=2 and T=14.56 . **(A)** Optimal stimulus calculated for the unconstrained case (blue), $u_{max}=0.3$ (red), and $u_{max}=0.2$ (green). **(B)** Time series traces of the phases $\theta_{1}$ (blue) and $\theta_{2}$ (red) of two neurons, where both have the optimal stimulus applied with $u_{max}=0.3$ . **(C)** Phase difference ϕ between two neurons over multiple stimuli. Each stimulus $u^{*}$ is triggered when $\theta_{1}=0$ . **(D)** Log of the difference ϕ over multiple stimuli. **(E)** Approximation to the Lyapunov exponent $\Lambda_{fit}$ found from the slopes of the fits to log(ϕ) versus t (red) and the Lyapunov exponent $\Lambda_{\mathrm{calc}}$ (blue), for different values of $u_{max}$ . **(F)** Effect of the constraint on the energy used for the optimal stimulus, here computed for one cycle of the stimulus. For panels **(B–F)**, the initial conditions are $\theta_{1}=0$ and $\theta_{2}=0.1$ .

**FIGURE 5:**
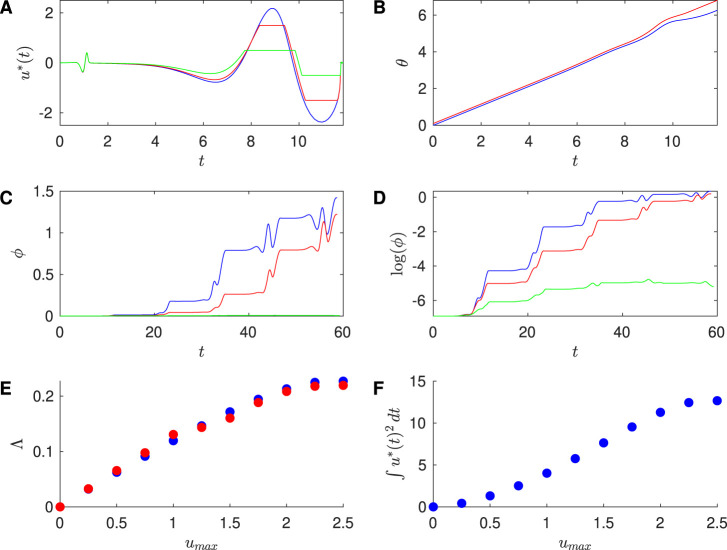
Results for Reduced Hodgkin-Huxley PRC with β=9 and T=11.85 . **(A)** Optimal stimulus calculated for the unconstrained case (blue), $u_{max}=1.5$ (red), and $u_{max}=0.5$ (green). **(B)** Time series traces of the phases $\theta_{1}$ (blue) and $\theta_{2}$ (red) of two neurons with initial conditions $\theta_{1}=0$ and $\theta_{2}=0.1$ , where both have the optimal stimulus applied with $u_{max}=1.5$ . **(C)** Phase difference ϕ between two neurons over multiple stimuli. Each stimulus $u^{*}$ is triggered when $\theta_{1}=0$ . **(D)** Log of the difference ϕ over multiple inputs. **(E)** Approximation to the Lyapunov exponent $\Lambda_{fit}$ found from the slopes of the fits to log(ϕ) versus t (red) and the Lyapunov exponent $\lambda_{\mathrm{calc}}$ (blue), for different values of $u_{max}$ . **(F)** Effect of the constraint on the energy used for the optimal stimulus, here computed for one cycle of the stimuilus. For panels **(C–F)**, the initial conditions are $\theta_{1}=0$ and $\theta_{2}=0.001$ .

Similarly, Figure 3 shows results for the SNIPER PRC with $Z_{d}=0.5$ , β=2 , and ω=1 , corresponding to T=2π , and Table 2 compares $\Lambda_{fit}$ and $\Lambda_{\mathrm{calc}}$ for several values of $u_{\max}$ . Moreover, Figure 4 shows results for the Hodgkin-Huxley PRC with β=2 and period T=14.56 , which was obtained numerically; Table 3 compares the Lyapunov exponent estimates. Finally, Figure 5 shows results for the Reduced Hodgkin-Huxley PRC with β=9 and period T=11.85 , which was obtained numerically; Table 4 compares the Lyapunov exponent estimates. In all of these examples, the optimal input gives exponential divergence of the phases of the neurons. As $u_{max}$ becomes smaller, the Lyapunov exponent becomes smaller while staying positive, and the energy associated with the input stimulus is reduced. We observe that the numerically calculated optimal inputs without the magnitude constraint resemble $Z^{\prime }\left(\theta \right)$ for each PRC. This was first noticed in Wilson and Moehlis (2014b), where it was attributed to the numerical observations that the optimal input is weak enough that θ∼ωt , and $\beta Z^{\prime }\left(\theta \right)$ dominates λZ(θ) in Equation 10, so $u^{*}\left(t\right)\approx \beta Z^{\prime }\left(\omega t\right)/2$ . This approximation is explored in more detail in Moehlis et al. (2025).

**TABLE 2:** For the SNIPER PRC, comparison of the Lyapunov exponent $\Lambda_{fit}$ estimated from slope of the line of best fit for log(ϕ) versus t with the Lyapunov exponent $\Lambda_{\mathrm{calc}}$ calculated from the integral formulation.

$u_{max}$	$\Lambda_{fit}$	$\Lambda_{\mathrm{calc}}$
0.5	0.122	0.123
0.35	0.103	0.101
0.2	0.0626	0.0618

**TABLE 3:** For the Hodgkin-Huxley PRC, comparison of the Lyapunov exponent $\Lambda_{fit}$ estimated from slope of the line of best fit for log(ϕ) versus t with the Lyapunov exponent $\Lambda_{\mathrm{calc}}$ calculated from the integral formulation.

$u_{max}$	$\Lambda_{fit}$	$\Lambda_{\mathrm{calc}}$
0.4	0.0239	0.0243
0.3	0.0221	0.0222
0.2	0.0163	0.0172

**TABLE 4:** For the Reduced Hodgkin-Huxley PRC, comparison of the Lyapunov exponent $\Lambda_{fit}$ estimated from slope of the line of best fit for log(ϕ) versus t with the Lyapunov exponent $\Lambda_{\mathrm{calc}}$ calculated from the integral formulation.

$u_{max}$	$\Lambda_{fit}$	$\Lambda_{\mathrm{calc}}$
2.5	0.219	0.227
1.5	0.160	0.172
0.5	0.0655	0.0625

Here we make the new observation that in some cases the magnitude-constrained optimal input resembles the unconstrained input simply “chopped off” at the constraint, i.e., $u_{chop}\equiv \min\left(u_{max},\max\left(-u_{max},\tilde{u}\left(t\right)\right)\right)$ , where here $\tilde{u}$ would be the optimal input found by solving Equations 7, 8 using Equation 10, i.e., without any magnitude constraint. That said, we note that $u_{chop}$ is not necessarily a good approximation to the optimal input. Solutions to the two-point boundary value problem have the property of modifying a neuron’s phase to go from θ=0 to $\theta =\omega t_{1}$ in the time $t_{1}$ . This must be the case for the unconstrained optimal input $\tilde{u}\left(t\right)$ and for the constrained optimal input $u^{*}\left(t\right)$ . Because $u_{chop}$ is different from $\tilde{u}\left(t\right)$ during the time intervals for which the constraint is applied, but otherwise the same, we do not expect it to exactly take θ from 0 to $\omega t_{1}$ in the time $t_{1}$ . However, numerically we find for some examples that $u^{*}\left(t\right)$ looks very similar to $u_{chop}$ . This appears to be because the product Z(θ)u(t) is very small in these examples. But, it is clear from Figure 5 that for the constraint $u_{max}=0.5$ the optimal $u^{*}\left(t\right)$ can be significantly different from $u_{chop}\left(t\right)$ .

**FIGURE 6:**
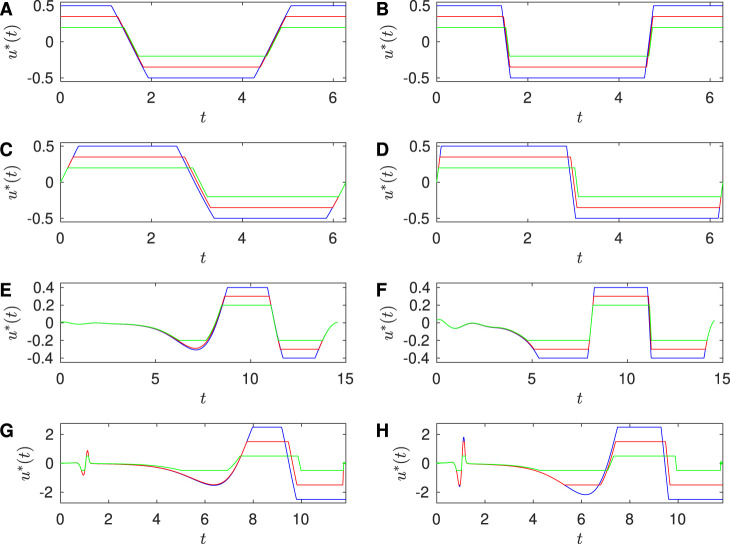
Approaching Bang-bang control in the large β limit. **(A)** Results for Sinusoidal PRC with $Z_{d}=0.5$ and ω=1 , for β=5 and **(B)** β=20 . Optimal stimulus calculated for $u_{max}=0.5$ (blue), $u_{max}=0.35$ (red), and $u_{max}=0.2$ (green). **(C)** Results for SNIPER PRC with $Z_{d}=0.5$ and ω=1 , for β=5 and **(D)** β=20 . Optimal stimulus calculated for $u_{max}=0.5$ (blue), $u_{max}=0.35$ (red), and $u_{max}=0.2$ (green). **(E)** Results for Hodgkin-Huxley PRC with β=5 and **(F)** with β=20 . Optimal stimulus calculated for $u_{max}=0.4$ (blue), $u_{max}=0.3$ (red), and $u_{max}=0.2$ (green); here T=14.56 . **(G)** Results for Reduced Hodgkin-Huxley PRC with β=20 and **(H)** with β=40 . Optimal stimulus calculated for $u_{max}=2.5$ (blue), $u_{max}=1.5$ (red), and $u_{max}=0.5$ (green); here T=11.85 .

When β is large, the Lyapunov exponent term in Equation 4 dominates the energy usage term. Therefore, in the limit of large β the optimal inputs approach what is known as Bang-bang control, as seen in Figure 6. For Bang-bang control, the controller alternates between maximum and minimum inputs which switch at optimal times (Kirk, 1998). In particular, the control is driven by the sign of $Z^{\prime }\left(\theta \right)$ : if $Z^{\prime }>0$ we take $u=u_{max}$ , and if $Z^{\prime }<0$ we take $u=-u_{max}$ . Thus, at every time the integrand is maximized subject to the $u_{max}$ constraint, so the value of the Lyapunov exponent is maximized. The unconstrained stimulus magnitudes rise with increasing β , so in the large β limit the magnitude constraint becomes more important. Figure 6 shows the approach to Bang-bang control for increasing β (from left to right panel for each row) on the optimal input for each PRC.

### 3.2 Results for population-level simulations of neurons

While the results in the previous section illustrate that the stimuli with and without magnitude constraints can give positive Lyapunov exponents for the phase difference betweeen pairs of neural oscillators, we are also interested in how such inputs perform for a larger population of coupled neural oscillators. In this section, we consider a population of Reduced Hodgkin Huxley neurons with all-to-all electrotonic coupling, and independent additive noise for each neuron. The governing equations are:

$$\begin{array}{ccc} {\dot{V}}_{i} & = & f_{V}\left(V_{i},n_{i}\right)+\frac{1}{N}\sum_{j=1}^{N}\alpha \left(V_{j}-V_{i}\right)+u\left(t\right)+\eta_{i}\left(t\right),\\ {\dot{n}}_{i} & = & f_{n}\left(V_{i},n_{i}\right), \end{array}$$

where N=100 is the number of neurons, u(t) is the common input for all neurons, α=0.04 is the coupling strength, and $\eta_{i}\left(t\right)=\sqrt{2D}N\left(0,1\right)$ is intrinsic noise modeled as zero-mean Gaussian white noise with variance 2D , where D=0.7 . These values are chosen so that there is a balance between the synchronizing influence of the coupling and the desynchronizing influence of the noise. Expressions for $f_{V}$ and $f_{n}$ are given in the Supplementary Appendix. We suppose that at t=0 all neurons have the same $V_{i}$ and $n_{i}$ corresponding to the neuron at the peak of its action potential. Each neuron receives the same input u(t) , but a different realization of noise $\eta_{i}\left(t\right)$ . It is useful to think of the noise as having a desynchronizing effect, and the coupling as having a synchronizing effect.

**FIGURE 7:**
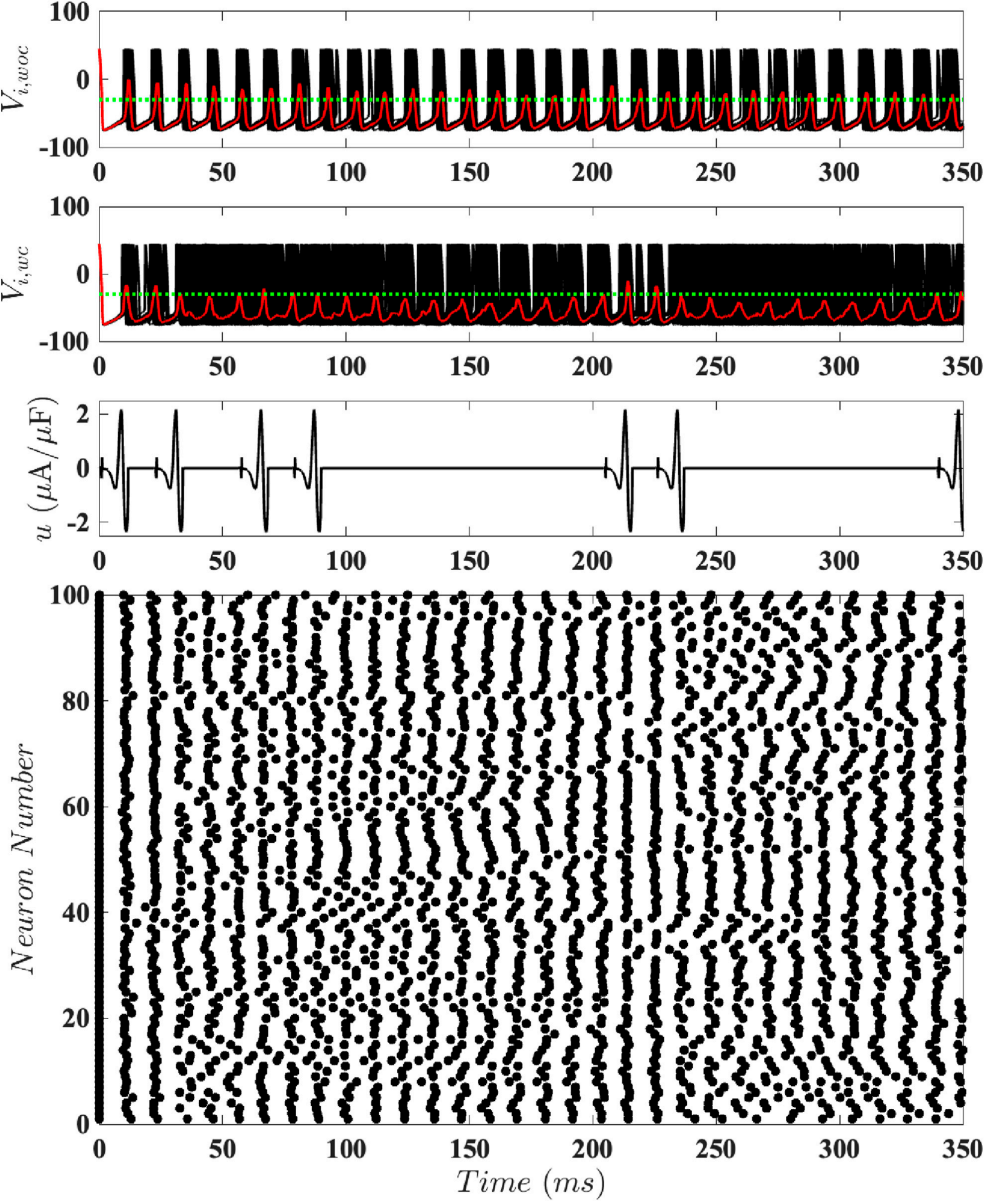
Results for Population-Level Simulations with Unconstrained Input. Here α=0.04 and D=0.7 . The top panel shows the network’s behavior without control, with synchronized dynamics. The second panel illustrates the same network with event-based control using the optimal u(t) without any magnitude constraints, where the red trace shows the mean voltage, and the horizontal green dotted line shows the control activation threshold ( $\bar{V}=-30$ mV). The third panel shows the control input. The bottom panel is a raster plot of the spike times.

**FIGURE 8:**
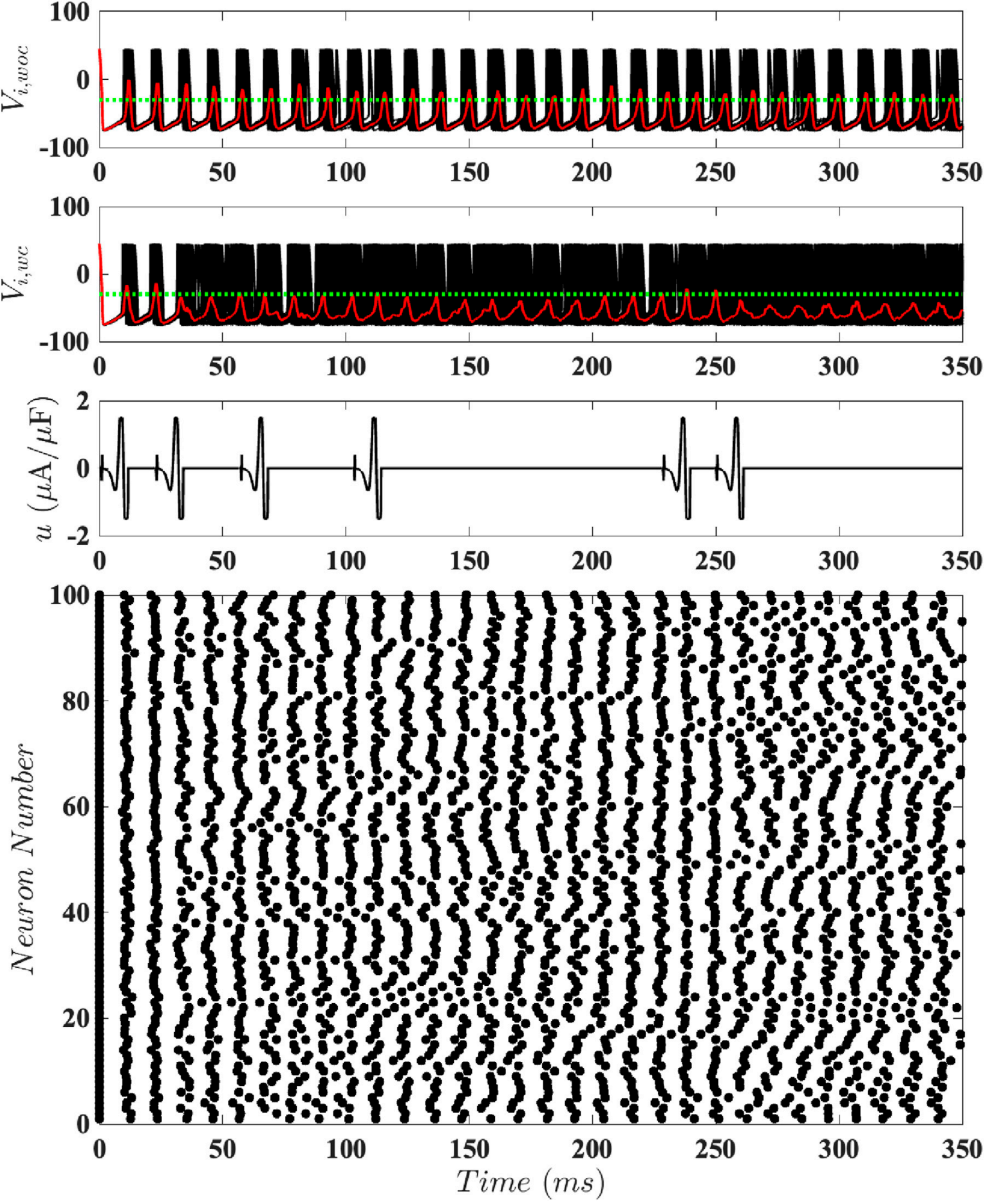
Results for Population-Level Simulations with Constrained Input at $u_{max}=1.5$ . Here α=0.04 and D=0.7 . The top panel shows the network’s behavior without control, with synchronized dynamics. The second panel illustrates the same network with event-based control using the optimal u(t) with $u_{max}=1.5$ , where the red trace shows the mean voltage, and the horizontal green dotted line shows the control activation threshold ( $\bar{V}=-30$ mV). The third panel shows the control input. The bottom panel is a raster plot of the spike times.

**FIGURE 9:**
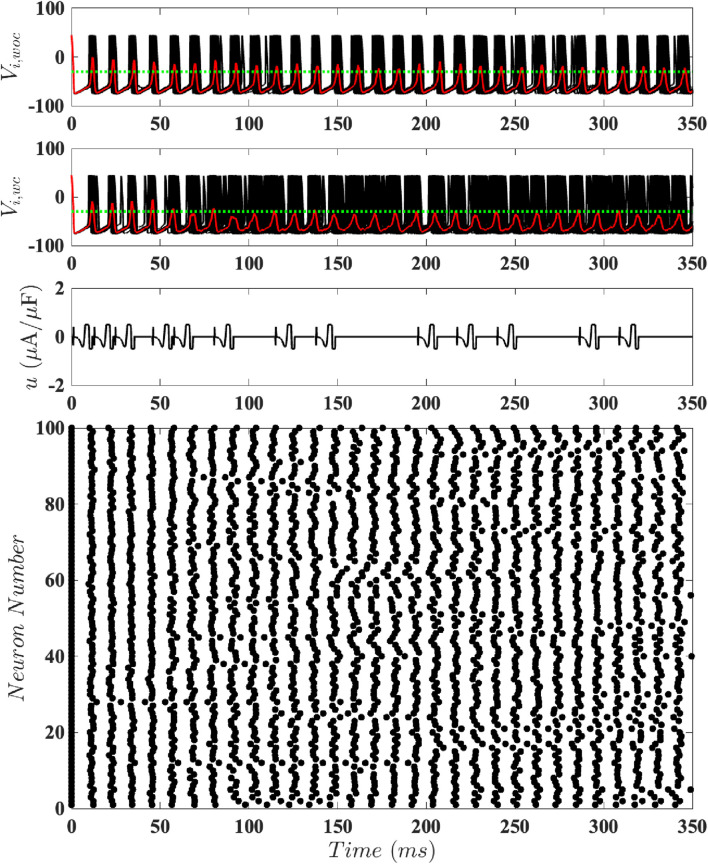
Results for Population-Level Simulations with Constrained Input at $u_{max}=0.5$ . Here α=0.04 and D=0.7 . The top panel shows the network’s behavior without control, with synchronized dynamics. The second panel illustrates the same network with event-based control using the optimal u(t) with $u_{max}=0.5$ , where the red trace shows the mean voltage, and the horizontal green dotted line shows the control activation threshold ( $\bar{V}=-30$ mV). The third panel shows the control input. The bottom panel is a raster plot of the spike times.

To test our magnitude-constrained optimal inputs $u^{*}\left(t\right)$ , we use an event-based control scheme similar to Danzl et al. (2009), Nabi et al. (2013), Wilson and Moehlis (2014b), Rajabi et al. (2025). In particular, when the average voltage $\bar{V}$ for the neurons crosses a threshold, we input one cycle of the pre-computed optimal stimulus, which will have a desynchronizing influence. Another control input occurs if the previous input has finished and the average voltage again crosses threshold, for example, due to the synchronizing influence of the coupling. According to this control logic, each simulation generates a control input u(t) for the full 350 msec time window. Figures 7–9 respectively show results for population-level simulations for the optimal unconstrained input, optimal constrained input with $u_{max}=1.5$ , and optimal constrained input with $u_{\max}=0.5$ . Comparing the results using event-based control with the network’s behavior without control, it is apparent that all of these stimuli are able to keep the neural population desynchronized. We can interpret these results as follows: if the population is too synchronized (its average voltage goes above the control activation threshold), a cycle of input is applied. If this does not sufficiently desynchronize the population, another cycle of input is applied. If the population is sufficiently desynchronized, no input is needed. However, the coupling eventually leads to a level of synchronization which is above the control activation threshold, triggering another cycle of input. For $u_{max}=0.5$ it is apparent that more cycles of the stimulus are needed to achieve and maintain desynchronized dynamics; see Figure 9.

**FIGURE 10:**
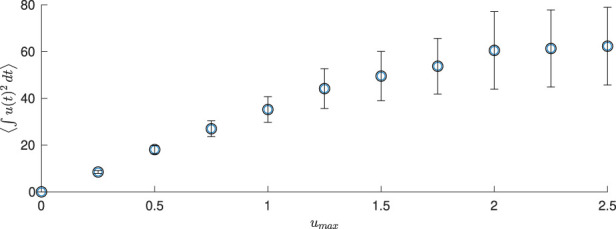
Energy used in population-level simulations for 350 msec for different values of $u_{\max}$ , averaged over 100 simulations. Here α=0.04 and D=0.7 .

This motivates us to better understand how the average amount of energy required to keep the neural population desynchronized with this event-based scheme depends on the magnitude constraint. To investigate this, we consider 100 different population-level simulations for different values of $u_{max}$ . For each simulation, the neurons start out completely synchronized. Results are shown in Figure 10 for the average energy

$$\left\langle \int_{0}^{350}{\left[u\left(t\right)\right]}^{2}dt\right\rangle ,$$

where ⟨⋅⟩ denotes the average over the 100 simulations, and the integration is over the first 350 msec . The error bars represent the standard deviation over the 100 iterations of the population-level simulations. The variability is due to the different realizations of noise for each simulation. We see that the average energy needed to desynchronize the population increases monotonically with $u_{\max}$ , until the constraint no longer has any effect on the computed input.

We used the average voltage as a measure of synchronization because this is easily defined, and one expects this to be related to the local field potential, which can be measured experimentally. However, a more common measure of synchronization is the Kuramoto order parameter (Kuramoto, 1984), whose amplitude is

$$R\equiv \left|\frac{1}{N}\sum_{j=1}^{N}e^{i\theta j}\right|,$$ (15)

where N is the number of neurons, and $\theta_{j}\in \left.\left[0,2\pi \right.\right)$ is the phase of the $j^{\text{th}}$ neuron in the sense of isochrons (Winfree, 1967). In particular, the phase is defined at all points in the basin of attraction of the periodic orbit; this is important because to calculate the Kuromoto order parameter we need to know the phase even if noise, coupling, and/or a control input causes the state of the neuron to be off the periodic orbit. We define θ=0 (which is equivalent to θ=2π ) to be the phase at which the neuron spikes, and parametrize the isochrons so that $\dot{\theta }=2\pi /T$ in the absence of noise, coupling, and control input, where T is the period of the neuron. We can estimate the phase of a neuron by setting its current state $\left(V_{i},n_{i}\right)$ as the initial condition at t=0 for the Reduced Hodgkin-Huxley equations for a single neuron in the absence of noise, coupling and control input. We integrate these equations forward in time until a voltage spike occurs at time $t_{s}$ . Because the spike corresponds to θ=2π and the phase advances according to $\dot{\theta }=2\pi /T$ , the phase corresponding to the state $\left(V_{i},n_{i}\right)$ is given by Equation 16:

$$\theta =2\pi \left(1-\frac{t_{s}}{T}\right).$$ (16)

To obtain the order parameter R at a given time, we estimate the phases of all N=100 neurons at that time, and use these in Equation 15. Higher values of R correspond to greater synchronization.

**FIGURE 11:**
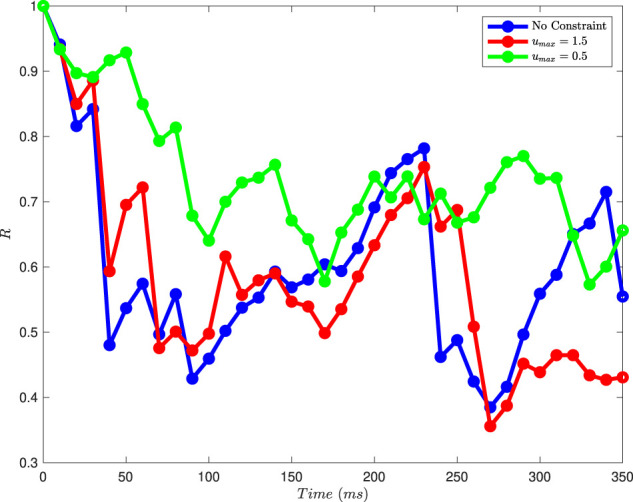
The magnitude of the Kuramoto order parameter for different constraints on the magnitude of the control input, calculated at 10 msec intervals for the results shown in Figures 7–9. Higher values of R correspond to greater synchronization.

Figure 11 shows results from this order parameter calculation for the plots shown in Figure 7 (no constraint on the magnitude of u ), Figure 8 (for $u_{max}=1.5$ ), and Figure 9 (for $u_{max}=0.5$ ), at 10msec intervals. We see that, broadly speaking, the control inputs tend to reduce R , and that R increases when there are no control inputs, due to the synchronizing effect of the coupling. The results for the no constraint case and for $u_{max}=1.5$ are quite similar except for later times. The fact that R increases more rapidly in the no constraint case than for $u_{max}=1.5$ is due to the variability in results due to noise. More interestingly, we see that the order parameter tends to have higher R values for $u_{max}=0.5$ ; because the control input is more constrained, a single cycle of input has a smaller effect on the order parameter.

## 4 Discussion

Motivated by deep brain stimulation treatment of neurological disorders including Parkinson’s disease, there has been much recent interest in using control theory to design optimal input stimuli which desynchronize neural populations. One such approach used chaotic desynchronization to achieve this goal in an energy-optimal fashion (Wilson and Moehlis, 2014b). In this paper, we generalized this optimal chaotic desynchronization methodology by including a magnitude constraint on the input. In particular, we showed how to calculate the optimal stimuli which satisfy such a contraint, and demonstrated that such inputs lead to exponential desynchronization of pairs of neurons and effective dysynchronization of populations of coupled, noisy neurons. This approach is based on a phase-reduction of the dynamics of a neuron, with the phase response curve quantifying the effect of an external input on a neuron’s phase. Based on the knowledge of a neuron’s phase response curve, we calculated the inputs that are optimally desynchronizing while minimizing energy utilization, using methods from optimal control. The addition of a magnitude constraint allows for the design of optimal stimulation inputs with a maximum amplitude that is customizable based on biological and electronic considerations. Interestingly, while these constrained inputs use less energy, they still achieve population-level desynchronization.

We note an extension of the current paper which could be of interest in future work: incorporation of both magnitude and charge-balance constraints on the control stimulus. This follows from the observation that non-charge-balanced stimuli, such as those considered in this paper, can cause harmful Faradaic reactions that may damage the DBS electrode or neural tissue (Merrill et al., 2005). This has motivated the use of a charge-balance constraint for optimal control design (Nabi and Moehlis, 2009; Wilson and Moehlis, 2014b). However, this presents additional challenges because it increases the dimension of the two-point boundary value problem which must be solved numerically. Our approach could be applied to other types of neurons, including those for common neurostimulation targets such as the subthalamic nucleus or the thalamus, even for periodically bursting neurons, as long as the phase response curve can be determined. If it is not possible to obtain this from electrophysiological measurements (Netoff et al., 2012), or if there is not an accurate mathematical model which would allow numerical techniques to be used Ermentrout (2002), Monga et al. (2019), an approach such as that described in Wilson and Moehlis (2015b), which can estimate the phase response curve based on aggregate measurements such as the local field potential, could be used. Moreover, we expect that similar population-level control results would be found for other types of coupling, such as synaptic coupling and/or heterogeneous coupling, provided that the coupling strength is not too strong.

We imagine that the results from this paper can be useful to the neuroscience community in cases where there are biological or electronic hardware considerations which limit the allowed input magnitude for a stimulus. With deep brain stimulation becoming an increasingly adopted therapeutic technique for treatment of neurological disorders, this research extends ongoing research efforts to theoretically inform the optimal design of deep brain stimulation protocols.

## Data Availability

The datasets presented in this study can be found in online repositories. The names of the repository/repositories and accession number(s) can be found below: (https://github.com/michaelzimet/MgOptChaoticDesync) (DOI: 10.5281/zenodo.15595745).
