# Supplementary material for: Magnitude-constrained optimal chaotic desynchronization of neural populations
Source: Front Netw Physiol. 2025 Oct 21;5:1646391. doi: 10.3389/fnetp.2025.1646391 (PMC12583030; doi:10.3389/fnetp.2025.1646391)
Supplement: Supplementary file 1 [file Supplementaryfile1.pdf]

SUPPLEMENTARY APPENDIX: NEURON MODELS

Hodgkin-Huxley Equations

The Hodgkin-Huxley equations are given by (Hodgkin and Huxley (1952)):

$$\begin{aligned}
\frac{dV}{dt} &= [I_b - \bar{g}_{Na}m^3h(V - V_{Na}) - \bar{g}_K n^4(V - V_K) - g_L(V - V_L)]/C,\\
\frac{dn}{dt} &= \alpha_n(V)(1-n) - \beta_n(V)n,\\
\frac{dm}{dt} &= \alpha_m(V)(1-m) - \beta_m(V)m,\\
\frac{dh}{dt} &= \alpha_h(V)(1-h) - \beta_h(V)h,
\end{aligned}$$

where

$$\begin{aligned}
\alpha_n(V) &= \frac{0.01(V+55)}{1-\exp[-(V+55)/10]}, \qquad \beta_n(V) = 0.125\exp[-(V+65)/80],\\
\alpha_m(V) &= \frac{0.1(V+40)}{1-\exp[-(V+40)/10]}, \qquad \beta_m(V) = 4\exp[-(V+65)/18],\\
\alpha_h(V) &= 0.07\exp[-(V+65)/20], \qquad \beta_h(V) = \frac{1}{1+\exp[-(V+35)/10]}.
\end{aligned}$$

Here V is the transmembrane voltage, I_b is the baseline current, and n, m, and h are dimensionless gating variables which have range $[0, 1]$. In these equations, voltages are measured in mV, current density in $\mu A/cm^2$, capacitance density in $\mu F/cm^2$, and time in $msec$. The canonical values of the constants are

$$\bar{g}_{Na} = 120\ mmho/cm^2, \qquad \bar{g}_K = 36\ mmho/cm^2, \qquad g_L = 0.3\ mmho/cm^2,$$

$$V_{Na} = 50mV, \qquad V_K = -77\ mV, \qquad V_L = -54.4\ mV, \qquad C = 1\ \mu F/cm^2.$$

Reduced Hodgkin-Huxley Equations

The Reduced Hodgkin-Huxley Equations are found by making the following approximations (Keener and Sneyd (1998); Moehlis (2006)): (i) the gating variable m evolves on a fast timescale and instantaneously takes the value found by solving $\frac{dm}{dt} = 0$ for the given value of the voltage, and (ii) $h(t) = 0.8 - n(t)$. This gives the following two-dimensional system of equations:

$$\frac{dV}{dt} = \{I_b - \bar{g}_{Na}[m_\infty(V)]^3(0.8-n)(V - V_{Na}) - \bar{g}_K n^4(V - V_K) - g_L(V - V_L)\}/C, \tag{1}$$

$$\frac{dn}{dt} = \alpha_n(V)(1-n) - \beta_n(V)n. \tag{2}$$

REFERENCES

Hodgkin, A. L. and Huxley, A. F. (1952). A quantitative description of membrane current and its application to conduction and excitation in nerve. *J Physiol.* 117, 500–544. doi:10.1113/jphysiol.1952.sp004764. 16

Keener, J. and Sneyd, J. (1998). *Mathematical Physiology* (New York: Springer)

Moehlis, J. (2006). Canards for a reduction of the Hodgkin-Huxley equations. *Journal of Mathematical Biology* 52, 141–153. doi:10.1007/s00285-005-0347-1
